# Application of rectal retractor for postprostatectomy salvage radiotherapy of prostate cancer: A case report and literature review

**DOI:** 10.1002/ccr3.2430

**Published:** 2019-09-27

**Authors:** Hamed Ghaffari, Mahdieh Afkhami Ardekani, Seyed Hadi Molana, Mohammad Haghparast, Mastaneh Sanei, Seied Rabi Mahdavi, Bahram Mofid, Aram Rostami

**Affiliations:** ^1^ Department of Medical Physics School of Medicine Iran University of Medical Sciences Tehran Iran; ^2^ Department of Radiology Faculty of Para‐Medicine Hormozgan University of Medical Sciences Bandare‐Abbas Iran; ^3^ Department of Radiation Oncology School of Medicine AJA University of Medical Sciences Tehran Iran; ^4^ Department of Radiation Oncology School of Medicine Iran University of Medical Sciences Tehran Iran; ^5^ Department of Radiation Oncology Shohada‐e‐Tajrish Medical Center Shahid Beheshti University of Medical Sciences Tehran Iran; ^6^ Department of Medical Physics Roshana Cancer Institute Tehran Iran

**Keywords:** IMRT, postprostatectomy, rectal dose, rectal retractor

## Abstract

Using a rectal retractor (RR) during salvage radiotherapy after radical prostatectomy is a promising approach for reducing dose to the rectum. The patient well tolerated the daily RR insertion. This area of research encourages researchers for a comprehensive evaluation of the role of the RR in postprostatectomy radiotherapy.

## Graphical abstract

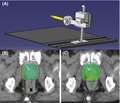

## 1 INTRODUCTION

This report aimed to introduce a rectal retractor (RR) application in postprostatectomy radiotherapy setting. A 74‐year‐old male, referred for salvage hypofractionated IMRT after prostatectomy for prostate cancer. Using a RR was feasible and significantly reduced rectal doses. Rectal retractor application is a novel promising approach to spare rectum in postprostatectomy radiotherapy.

Radical prostatectomy (RP) is considered as a state of the art curative treatment modality in the management of localized prostate cancer. However, up to 1/3 prostate cancer patients experience the biochemical relapse that salvage radiotherapy (RT) has been shown to improve treatment outcomes for patients with prostate cancer relapsing after RP. As demonstrated in many randomized clinical trials, there is a dose‐response relationship for the salvage RT similar to the definitive RT. Five‐year biochemical relapse‐free survival improved from 25% to 58% when total prescription dose increased from 60 to 70 Gy.^1^ However, one of risks associated with dose‐escalated prostate RT is rectal toxicity that has moderate or severe effect on patient's quality of life.

Intensity‐modulated radiation therapy (IMRT) and image‐guided radiation therapy (IGRT) have led to better sparing surrounding normal tissue, dose conformity, and also reducing planning target volume (PTV) margin.^2^, ^3^ Assuming that the α/β ratio in prostate cancer is close to 1.5 Gy and yet lower than the bladder and the rectum, hypofractionated RT can provide a theoretical biological benefit over conventional RT regimens.^4^ However, rectal tolerance can be the main limitation factor to expanded application of hypofractionated prostate RT. Previous study by our group has shown that a rectal retractor (RR) can significantly reduce rectal wall doses during definitive prostate RT. Also, the retraction of the rectum resulted in a significant anterior rectal wall dose reduction. The use of the RR is well tolerated by patients with minimal anal irritation.^5^, ^6^ To our knowledge, the effect of a RR on the rectal doses or toxicity in postprostatectomy RT has not been evaluated. Herein, we introduce a RR and report its efficacy in postprostatectomy salvage hypofractionated IMRT. Furthermore, we also summarize the role of different rectal displacement devices such as hydrogel rectal spacers and endorectal balloons (ERBs) in postprostatectomy RT setting.

## 2 CASE PRESENTATION

A 74‐year‐old male underwent radical prostatectomy in March 2013. In May 2016, PSA level was 0.18 ng/mL. However, in November 2017 his PSA level increased to 1.64 ng/mL. Therefore, local disease recurrences were diagnosed on magnetic resonance imaging (MRI). Two 14 × 18 mm and 9 × 8 mm lesions were seen at right periurethral space and right anterior aspect of vesicourethral anastomotic site, respectively. Both of mentioned lesions showed moderate signal intensity in T2‐weighted MRI, with abnormal choline/citrate ratio in magnetic resonance spectroscopy (MRS) study. No abnormal pelvic lymph adenopathy was present.

**Figure 1:**
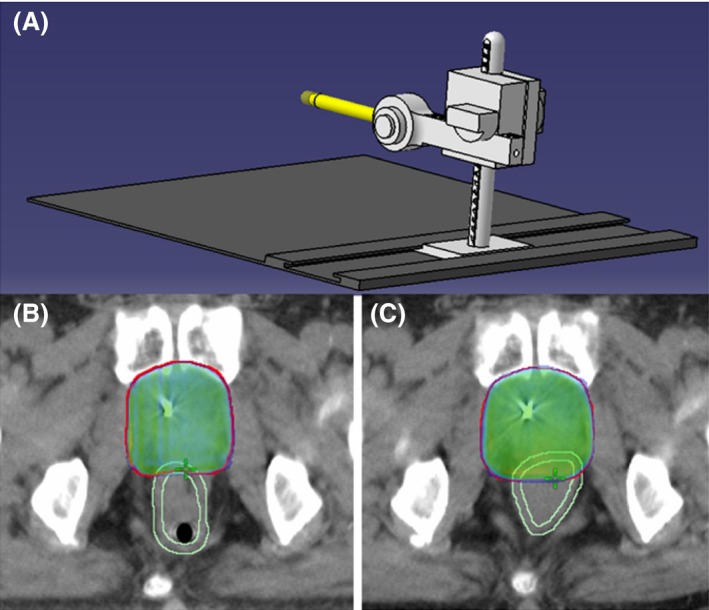
Three‐dimensional mechanical drawing of the rectal retractor system (A), transversal view of plans for CT‐scans with rectal retractor in‐place (B) and without rectal retractor (C)

We previously developed a RR in‐house and treated prostate cancer patients with dose‐escalated RT (40 × 2 Gy) with RR in‐place.^5^, ^6^ As shown in Figure 1A, the RR involves a Perspex rod that is inserted into the rectum to extend beyond the middle of the seminal vesicles in the supine or decubitus position. The shaft of the rectal rod is then connected to a vertical locking column attached to a carbon‐fiber baseplate. The rectal rod is depressed to gradually displace the rectal wall dorsally away from the prostate gland. The patient's comfort and tolerability provide a guide for the rectal retraction. The vertical depression of the RR system in the planning computed tomography (CT) is reproduced for daily treatment sessions.

Three‐gold fiducial markers were implanted in the prostate bed. Five days after seed implantation, the patient underwent two planning CT‐scans at 3 mm slice thickness in the supine position, one without RR and then one with RR. For hygienic reasons, the rectal rod was covered by disposable condom. The rectal rod insertion was facilitated by adequate lidocaine jelly. The patient was asked to empty the rectum and bladder and then drinking 500 cc of water for achieving a comfortable full bladder 30 minutes before planning CT and each RT session.

The CT‐images were imported into the Varian Eclipse v.13.6 (Varian Medical System Inc.) treatment planning software (TPS) for IMRT treatment planning. The prostate bed, seminal vesicles, rectal wall, anterior rectal wall, bladder, and femoral heads were delineated on the CT‐datasets. The prostate bed and 2/3 proximal seminal vesicles were defined as the clinical target volume (CTV) and a safety isotropic margin of 5 mm was added to for the PTV. The rectal wall was outlined from the anal canal to the rectosigmoid flexture. It should be noted that the volume of rectal wall is constant with and without RR.^7^ In addition, the anterior rectal wall was delineated, as we previously reported.^5^

Two similar plans were created, one without RR and one with RR. IMRT was delivered using a 7‐field to a total dose of 70.2 Gy in 26 fractions with a 6 MV photons. Prior to radiation delivery, online correction of the gold seeds was performed by electronic portal imaging device. The patient was treated using a RR in‐place.

## 3 RESULTS

**Figure 2:**
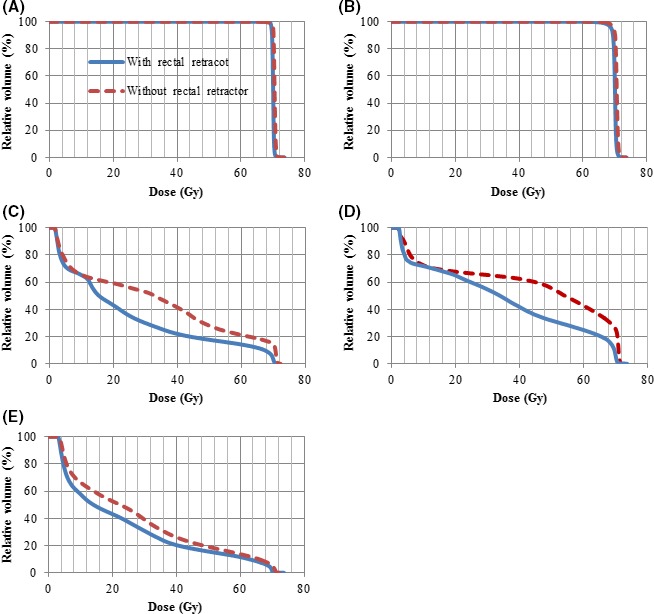
Dose‐volume histogram of the clinical target volume (A), planning target volume (B), rectal wall (C), anterior rectal wall (D), and bladder (E) that red line is without rectal retractor and blue line is with rectal retractor

Using a RR increased the distance between recurrence regions and lateral and posterior rectal wall. The CTV volumes were 57.2 cc without RR and 56.4 cc with RR. The anatomic and dosimetric characteristics of the PTV in plan with and without RR are shown in Table 1 and Figure 2B. The rectal wall and anterior rectal wall dose metrics were reduced in the plan with RR, as shown in Table 1 and Figure 2C,D. The use of the RR resulted in an absolute reduction of 8.5 Gy in the mean dose to the rectal wall (rectal wall Dmean). The relative reduction of the rectal wall V40 Gy, V50 Gy, V60 Gy, and V70 Gy were 47.2%, 37.1.6%, 33.5%, and 76.2%. The RR had a minimal effect on low and intermediate bladder dose distribution (Figure 2E). The anatomy before and after RR application are shown in Figure 1B,C.

**Table 1:** Dosimetric parameters on planning target volume and rectal wall with and without rectal retractor

	With RR	Without RR
PTV		
Volume (cc)	108.8	107.2
D_mean_ (Gy)	69.9	70.5
D_max_ (Gy)	73.7	73.5
V_95%_ (%)	99.0	99.8
D_95%_ (Gy)	69.0	69.6
Rectal wall		
Volume (cc)	32.8	31.4
D_mean_ (Gy)	23.9	32.4
V_40Gy_ (%)	21.9	41.5
V_50Gy_ (%)	17.6	28.0
V_60Gy_ (%)	14.1	21.2
V_70Gy_ (%)	3.5	14.7
V_60Gy_ (cc)	4.6	6.6
V_70Gy_ (cc)	1.1	4.6

Abbreviations: PTV, planning target volume; RR, rectal retractor.

PSA level was 1.53 ng/mL 1 week before the end of the RT sessions, no change in comparison with the baseline PSA level 1.64 ng/mL before the treatment. Two and 5 months after the end of RT treatment, the patient's PSA level remarkably decreased to 0.009 ng/mL and 0.007 ng/mL, respectively.

After 4 weeks of the treatment, the patient reported a mild degree of anal irritation (grade‐1 toxicity according to the Common Terminology Criteria for Adverse Events [CTCAE] v.4.0 classification) due to daily insertion of the rectal rod, but did not discontinue the rectal rod insertion in following treatment sessions. This degree of anal irritation resolved approximately 1 week after the end of RT. Based on our experience, this anal irritation can be well managed by using an adequate lidocaine jelly and lubrication gel. Adverse events such as rectal bleeding and severe anal irritation did not occur due to daily utilization of the RR. The patients experienced some local pressure on the posterior rectal wall after the rectal retraction, and this degree of pressure approximately resolved after 1‐2 minutes. However, there was a low degree of pressure during treatment session. Also, the patient felt a slight degree of pressure about 2 minutes after removing the rectal rod. The patient did not have rectal complications at the baseline (ie, beginning of RT). During the last week of treatment, the patient reported soft stools about once a day with occasional urgency (no need for medication)—grade‐1 rectal urgency according to CTCAE v.4.0 criteria. In the fifth week of treatment, the patient reported urinary urgency that was resolved with a spasmolytic anticholinergic agent, corresponding to grade‐2 urinary urgency according to CTCAE v.4.0 criteria. The patient did not report urinary or rectal grade 3 or 4 toxicities during the treatment or the following 5 months.

## 4 DISCUSSION

This report has been evaluated the impact of the RR on the rectal wall doses and acute rectal toxicities during postprostatectomy IMRT. To our knowledge, this is the first report that evaluated the impact of the RR on rectal wall doses during postprostatectomy salvage hypofractionated IMRT. Our data showed that using a RR reduced considerably rectal wall doses, which can be explained by pushing lateral and posterior rectal wall away from the PTV, as well as stretching rectal wall that reduces thickness of the rectal wall in high‐dose region. These results are in concordance with reports on the RR application in definitive prostate RT.^5^, ^6^, ^7^ The current report suggests that a RR can be helpful in dose‐escalated postprostatectomy hypofractionated IMRT. Meanwhile, safe acute rectal toxicity profiles have observed using RR. Besides, the RR may also offer others advantages in postprostatectomy RT such as dose escalation, the reproducibility of the rectum position, in vivo rectal wall dosimetry with passive or active dosimeters.

In addition to the RR, endorectal balloons (ERBs) and prostate‐rectum spacers have also used during definitive prostate RT, resulting in an improvement in rectal dosimetry and toxicity.^8^ In postprostatectomy salvage RT, a case report has been shown that the hydrogel spacer (SpaceOARTM System; Augmenix Inc, Waltham, Mass.) have been reduced the rectal doses during IMRT to a total dose of 76 Gy in 38 fractions. Acute toxicities were similar to the current report. However, authors reported that this method can be used in specifically selected patients.^9^ A recent study has shown that using a hydrogel rectal spacer is well tolerated by patients in postprostatectomy RT. This single‐arm study has reported that a minimal improvement in 5 years late GI and GU toxicities were achieved by using a hydrogel spacer.^10^ However, there were several limitations in that study such as single arm, retrospective investigation, using physician scoring to measure adverse events, and heterogeneous cohort. In the setting of biochemical failure and macroscopic local recurrence, the CTV should be included the entire prostatic fossa (the pubic bone to the rectal wall in anterior‐posterior axis plus potential microscopic tumor spread). Thus, hydrogel spacer application is not useful. The prostate‐rectum spacer can be used when there is only one macroscopically visible lesion that is responsible for local recurrence in very high level of confidence and safety. Also, the spacer injection or implantation can be led to the risk of potential tumor cell shift.^11^

Recently, Streller et al investigated the effect of an ERB on anorectal doses in postprostatectomy salvage volumetric‐modulated arc therapy that the application of an ERB did not result in a significant reduction of anorectal doses.^12^ Also, other reports on effect of an ERB in postoperative RT for prostate cancer showed that the ERB application led to an improvement in the anal wall doses and geometric reproducibility of the CTV, rectum, and bladder, but the dosimetric stability of the rectum with ERBs were controversial, which can be attributed to pushing anterior rectal wall toward the prostate bed by ERBs.^13^, ^14^ Missing counterfort of the prostate gland in the postoperative setting results in pushing anterior rectal wall into the treatment field by using an ERB more than the definitive prostate RT with an ERB. Besides, the cost of the ERBs and hydrogel rectal spacers is high,^15^ whereas the RR is cost‐effectiveness, and using a RR can reduce both cost and surgical risk associated with hydrogel spacer implantation. The application of ERB can result in increasing dose to the anterior rectal wall.^16^ Contrast to ERB, the use of the RR did not result in increasing dose to the anterior rectal wall, as shown in Figure 2D. Moreover, the ERB position is not reproducible between treatment sessions that can cause deformations in the prostate.^17^ As reported by studies, the implantation of prostate‐rectum spacers can lead to rectal ulceration, and perforation, perineal abscess, etc.^18^, ^19^, ^20^ Of note, rate of these complications is very low. Taken all together, RDDs can be helpful in postoperative dose‐escalated RT for prostate cancer, and RDDs may also reduce long‐term rectal toxicities.

Previous studies in definitive prostate RT with a RR in‐place showed that the RR increased the reproducibility of the rectum position.^5^, ^21^ Also, studies have been investigated the impact of a RR on the intrafraction prostate motion during VMAT using cone‐beam CT (CBCT) and kilovoltage intrafraction monitoring (KIM). As demonstrated in these studies, intrafraction prostate motion was reduced by a RR.^22^, ^23^ Thus, advantages in terms of low cost, rectal dose sparing, reproducibility of the rectal wall position, and prostate stabilization can be achieved with RR.

In conclusion, the use of a RR in the postprostatectomy salvage hypofractionated IMRT resulted in reducing dose to the rectal wall. The patient well tolerated the daily RR insertion. Hypofractionated IMRT with RR can be delivered with a safe acute toxicity profile. RR application is a novel promising approach to spare rectum in postprostatectomy RT. Large studies will be required to evaluate clinical benefits of RR in postoperative RT for prostate cancer.

## CONFLICT OF INTEREST

None declared.

## AUTHOR CONTRIBUTIONS

HGh: collected the data, wrote the manuscript, and critically revised the manuscript for important intellectual content. MAA and AR: collected the data. SHM: was responsible clinician. SHM, MH, and MS: made substantial contributions to conception and design. SRM and BM: responsible for the integrity of the work as a whole.

## ETHICAL APPROVAL

The study was approved by the ethics committee of Iran University of Medical Sciences, Tehran, Iran. Ethics No. is IR.IUMS.FMD.REC.1396.9411338003.

## RESEARCH INVOLVING HUMAN PARTICIPANTS AND/OR ANIMALS

This study involved human participant, and it was conducted considering ethic responsibilities according to the World Medical Association and the Declaration of Helsinki.

## INFORMED CONSENT

Informed consent was obtained from patient prior to his inclusion in the study.
